# Prebiotics, Probiotics and Nutrients in Cardiovascular and Kidney Disease

**DOI:** 10.3390/nu15194284

**Published:** 2023-10-08

**Authors:** Zitong Lei, Menglu Xu, Ying Li, Lei Chen, Hongbao Li

**Affiliations:** 1 Department of Critical Care Nephrology and Blood Purification, The First Affiliated Hospital of Xi’an Jiaotong University, Xi’an 710061, China; 2 Department of Nephrology, The First Affiliated Hospital of Xi’an Medical University, Xi’an 710077, China; 3 Department of Physiology and Pathophysiology, School of Basic Medical Sciences, Xi’an Jiaotong University, Xi’an 710061, China

Cardiovascular disease (CVD) and chronic kidney disease (CKD) are the leading causes of mortality and health burden worldwide. Emerging evidence suggests that the gut microbiota plays an important role in the development and progression of CVD and CKD [1,2]. One of the dysbiotic hallmarks in CVD and CKD is the imbalanced gut microbiota with the depletion of short-chain fatty-acid-producing bacteria and an increase in uremic-toxin-producing pathobionts. The accumulation of uremic toxins deteriorates multiple organs, especially the vascular system, heart, and kidney.

Recent investigations, including one review article of this Special Issue [3], have demonstrated the potential positive effect of different prebiotics, probiotic strains, or nutrients (e.g., potassium, omega-3 fatty acids, vitamin D, protein, methylfolate, functional foods) on the pathogenic mechanisms involved in CVD and CKD, including the modulation of inflammatory and immune responses, decrement of uremic toxins, and enhancement of the intestinal barrier function, in addition to a beneficial impact on gut homeostasis and dysbiosis [4,5]. One of the articles of this Special Issue shows that a proinflammatory dietary pattern is related to a higher risk of developing peripheral arterial disease (PAD) among US adults, indicating that a proinflammatory diet might lead to a higher risk of PAD [6].

Maternal–fetal crosstalk has been implicated in the long-term control of offspring health, including transgenerational hypertension. Previously, we found that the gut–brain axis via the mother may be a viable strategy for the control of hypertension in offspring [7]. Regarding targeting the gut–brain axis, dietary therapy is the most common strategy to modulate gut microbiota. Short chain fatty acids (SCFAs) have shown great potential in the treatment of microbial dysbiosis and have beneficial effects on hypertension. However, the mechanism by which SCFA-sensing receptors regulate blood pressure remains to be elucidated. In an article of the Special Issue [8], the authors explored dysregulated tissue levels of SCFAs and the expression of SCFA-sensing receptors in the hypothalamic paraventricular nucleus (PVN), a key forebrain region engaged in the neural regulation of blood pressure in offspring with maternal high fructose diet (HFD) exposure. They found that maternal HFD during gestation and lactation significantly reduced circulating butyrate, along with a decreased tissue level of butyrate and increased expression of SCFA-sensing receptors, GPR41 and olfr78. Tissue oxidative stress and neuroinflammation in the PVN of HFD offspring were rectified by oral supplements with biotics (prebiotics, probiotics, synbiotics, and postbiotics). The gene silencing of GPR41 or solfr78 mRNA in PVN protected adult HFD offspring from hypertension and alleviated the induced oxidative stress and inflammation in PVN. In addition, oral supplements with postbiotic butyrate restored tissue butyrate levels, rectified the expressions of GPR41 and olfr78 in PVN, and protected against programmed hypertension in adult HFD offspring.

Spermidine (SPD), a naturally occurring polyamine, which is one of the metabolites of gut microbiota that maintains vascular function, can also be used to explore its potential regulatory effects on the gut microbiota through dietary interventions. In another article of the Special Issue [9], the authors uncovered altered gut microbiota profiles and functions in Abdominal aortic aneurysm (AAA), with increased Bacteroides and Parabacteroides and reduced Prevotella, Desulfovibrionaceae, as well as the dysregulation of the biosynthesis and transportation of SPD. Exogenous SPD remitted the gut microbiota dysbiosis and attenuated the progression of AAA, making it possible to offer a cost-effective and reasonable alternative therapy for AAA and other diseases.

Regarding probiotics, several clinical studies have been conducted to determine the cholesterol-lowering effects of probiotic supplements in people with hypercholesterolemia [10]. Lactobacillus paracasei showed a potential effect in reducing cholesterol levels and preventing atherosclerosis [11,12]. In another article of this Special Issue [13], the researchers exhibited a clinical trial including 50 participants with hypercholesterolemia. They were randomly and equally assigned to consume L. paracasei TISTR 2593 or a placebo in maltodextrin capsules daily. Interestingly, the results demonstrated that supplementation with L. paracasei TISTR 2593 capsules for 90 days has a lowering effect on LDL-C, anti-oxidative stress, and anti-inflammation in Thai adults with high cholesterol levels. Thus, L. paracasei TISTR 2593 could be an adjuvant probiotic supplement to help manage LDL-C levels and potentially delay the development of atherosclerosis. It would be of interest to further explore the underlying mechanism of how L. paracasei TISTR 2593 exerts the reduction in blood lipids and prevents the development of atherosclerosis, such as the connection with the gut microbiota community, which may support probiotic-based food supplementations for managing hypercholesterolemia associated with cardiovascular diseases.

Similarly, dietary interventions including prebiotics, probiotics and nutrients serve as the first-line treatment for CKD; they affect the development of kidney disease through the gut–kidney axis. A review in the Special Issue [14] showed that certain prebiotic-rich diets, such as resveratrol and garlic oil, have demonstrated protective efficacy against CKD in children [15]. In addition, fecal microbiota transplantation [16] and colon dialysis [17] have been shown to be effective in restoring the diversity and structure of intestinal microbiota in children with kidney disease.

One of the articles of this Special Issue [18] designed and implemented a multicentric, double-blinded, randomized controlled trial of enzobiotic therapy (synbiotics and proteolytic enzymes) conducted over 12 weeks in 2019. The study evaluated the efficacy and safety of enzobiotics in reducing the generation of p-cresol sulfate (PCS) and indoxyl sulfate (IS), stabilizing renal function, and improving the quality of life (QoL). It also evaluated the feasibility of the diagnostic prediction of IS and PCS from CKD parameters. The study shows that enzobiotic therapy can potentially maintain renal function (GFR) and increase red blood cell and platelet count by controlling the generation of IS and PCS. In our previous research, we uncovered a novel beneficial role of F prausnitzii probiotics in the restoration of renal function in CKD by using a CKD mouse model, which is, at least in part, attributed to the butyrate-mediated GPR-43 signaling in the kidney, and provides the necessary foundation to harness the therapeutic potential of F prausnitzii for ameliorating CKD [19].

Resistant starch (RS) is a dietary fiber that alters the gut microbial consortium and leads to an increase in the microbial production of SCFAs, which could attenuate inflammatory and oxidative stress pathways, to reduce the progression of diabetic kidney disease (DKD), one of the leading causes of CKD. One review of the Special Issue [20] evaluated and summarized the evidence from both preclinical models of DKD and clinical trials that have utilized RS as a dietary therapy to limit the progression of DKD. Utilizing RS in early-stage DKD patients may present a cost-effective, non-medication-based tool to assist in attenuating disease development and progression. RS has the potential to affect several mechanisms implicated in DKD progression through its capacity to increase SCFA production, improve intestinal barrier integrity, downregulate inflammatory pathways and restore a healthy gut microbiome. 

Finally, Immunoglobulin A nephropathy (IgAN) is the leading cause of end-stage renal disease (ESRD) [21]. Identifying the specific gut microbiota and metabolites that have a causal relationship with IgAN remains challenging due to the complexity of the human gut microbiota. One of the articles of this Special Issue [22] employed Mendelian randomization (MR) to investigate the causal association between the gut microbiota and IgAN, examining a total of 15 metabolites and 211 microorganisms. Clinical specimens demonstrated the effectiveness and accuracy of Actinobacteria in distinguishing IgAN patients from those with other glomerular diseases, revealing a potential association between Actinobacteria abundance and increased albuminuria and a poorer prognosis in IgAN patients. This finding could provide valuable biomarkers for an early, noninvasive detection of the disease and potential therapeutic targets in IgAN.

The studies featured in this Special Issue are important in furthering the exploration and application of prebiotics, probiotics, and nutrients. As researchers continue to deepen their understanding of the gut microbiota, the treatment means of targeting the gut microbiota continue to evolve. Complex diet therapy can provide a variety of theoretical support for a better prognosis of CVD and CKD, but more clinical trials are needed to assess its therapeutic potential.
